# Chemical Evolution of Life on Earth

**DOI:** 10.3390/genes16020220

**Published:** 2025-02-13

**Authors:** Lei Lei, Zachary Frome Burton

**Affiliations:** 1 School of Biological Sciences, University of New England, Biddeford, ME 04005, USA; llei@une.edu; 2 Department of Biochemistry and Molecular Biology, Michigan State University, East Lansing, MI 48824, USA

**Keywords:** origin of life, chemical evolution, tRNA, minihelices, pre-life, last universal common (cellular) ancestor, anticodon, genetic code, type II tRNA, polyglycine

## Abstract

**Background/Objectives**: The origin of genes and genetics is the story of the coevolution of translation systems and the genetic code. Remarkably, the history of the origin of life on Earth was inscribed and preserved in the sequences of tRNAs. **Methods**: Sequence logos demonstrate the patterning of pre-life tRNA sequences. **Results**: The pre-life type I and type II tRNA sequences are known to the last nucleotide with only a few ambiguities. Type I and type II tRNAs evolved from ligation of three 31 nt minihelices of highly patterned and known sequence followed by closely related 9 nt internal deletion(s) within ligated acceptor stems. The D loop 17 nt core was a truncated UAGCC repeat. The anticodon and T 17 nt stem-loop-stems are homologous sequences with 5 nt stems and 7 nt U-turn loops that were selected in pre-life to resist ribozyme nucleases and to present a 3 nt anticodon with a single wobble position. The 7 nt T loop in tRNA was selected to interact with the D loop at the “elbow”. The 5′-acceptor stem was based on a 7 nt truncated GCG repeat. The 3′-acceptor stem was based on a complementary 7 nt CGC repeat. In pre-life, ACCA-Gly was a primitive adapter molecule ligated to many RNAs, including tRNAs, to synthesize polyglycine. **Conclusions**: Analysis of sequence logos of tRNAs from an ancient Archaeon substantiates how the pre-life to life transition occurred on Earth. Polyglycine is posited to have aggregated complex molecular assemblies, including minihelices, tRNAs, cooperating molecules, and protocells, leading to the first life on Earth.

## 1. Introduction

There is an overarching concept in the pre-life chemical evolution of biological systems that has perhaps been neglected or incompletely expressed. That is, biological systems initially evolved around a small number of central functional cores. For transcription systems, the central cores were two double-Ψ–β-barrel type DNA-dependent RNA polymerases, promoters, and TFBs in Archaea and σ factors in Bacteria [1,2]. TFBs and σ factors are homologs of one another made up of repeating helix–turn–helix motifs. Translation systems, by contrast, evolved around transfer RNA (tRNA), which is the genetic adapter. Essentially, all life and genetic coding evolved around the functional core of tRNA.

The evolution of life on Earth required chemical coevolution of tRNAomes (all the tRNAs of an organism), aminoacyl-tRNA synthetases (AARS), ribosomes, mRNA, and a genetic code, plus numerous interacting systems and molecules. This raises the question of how chemical evolution was driven by sufficiently powerful selective pressures before true cells could coalesce and commence Darwinian selection and evolution. This report addresses the question based on a history of chemical selection, written and recorded in conserved genetic code. Notably, the most central history of the pre-life-to-life transition on planet Earth was recorded in the sequences of tRNAs. Thus, the core story of the genesis of life on Earth was chronicled in tRNA sequences.

The three 31 nt minihelix tRNA evolution theorem shows how tRNA arose chemically on pre-life Earth [3,4]. For replication, a 31 nt D loop minihelix of known sequence was ligated to two 31 nt anticodon/T loop minihelices of almost entirely known sequence. After processing by 9 nt internal deletion(s) within ligated acceptor stems, type II and type I tRNAs were generated. Type II tRNA was formed by a single 9 nt internal deletion in a 93 nt tRNA precursor that was a replication intermediate for 31 nt minihelices. Type I tRNA was generated by two closely related 9 nt internal deletions in the same 93 nt tRNA precursor. The two internal 9 nt deletions to form type I tRNA were identical to one another on complementary RNA strands. An early version of type II tRNA could have been processed to type I tRNA by a single 9 nt internal deletion. Thus, the process for the generation of the first tRNAs was highly ordered and not chaotic or random, showing that the early Earth was capable during pre-life of ordered, chemically evolved processes. We posit that these ordered processes won out over random processes in chemical evolution because ordered processes were faster to establish core functions. Chaotic processes were simply too slow to generate the genetic adapter that is the core feature of life.

Remarkably, the tRNA body was initially made up of 100% RNA repeats and inverted repeats (stem-loop-stems) of known sequences. The 5′-acceptor stem was a 7 nt GCG repeat (GCGGCGG). The 3′-acceptor stem was a 7 nt complementary CGC repeat (CCGCCGC). The 17 nt D loop minihelix core was a UAGCC repeat (UAGCCUAGCCUAGCCUA). The 17 nt anticodon and T stem-loop-stems were initially of the sequence ~CCGGG_CU/???AA_CCCGG (_ separates stems and loops; / indicates a U-turn in the RNA backbone; ? indicates that, because of coding, the pre-life sequence is not now known). It is possible that the T loop minihelix was formed from the complement of the anticodon loop minihelix, with the initial sequence ~CCGGG_UU/???AG_CCCGG. The 9 nt internal deletion processing events were within ligated 3′- and 5′-acceptor stems (CCGCCGC_GCGGCGG→GGCGG (D 3′-stem and tRNA-26) and CCGCCGC_GCGGCGG→CCGCC (type I V loop)). These 9 nt internal deletions were identical on complementary RNA strands, so a single processing mechanism can account for both 9 nt internal deletions in type I tRNA. The type II V arm for tRNA^Leu^ and tRNA^Ser^ evolved from the initial sequence CCGCCGC_GCGGCGG to form a stem-loop-stem that could be discriminated accurately by LeuRS-IA (5 tRNA^Leu^ in a synonymous set) and SerRS-IIA (4 tRNA^Ser^ in a synonymous set) [5]. Leucine and serine occupy 6-codon sectors in the genetic code. Because there are 5 tRNA^Leu^ and 4 tRNA^Ser^ anticodons, the consistent and conserved tRNA type II V arms for each synonymous set were used as a determinant for tRNA^Leu^ and tRNA^Ser^ discrimination rather than the diverse anticodon loops. The number of synonymous type II tRNA sets in an organism is limited by the available trajectory set points of the long V arm: two set points in Archaea (tRNA^Leu^ and tRNA^Ser^) and three set points in Bacteria (tRNA^Tyr^, tRNA^Leu^ and tRNA^Ser^) [5]. From analysis of tRNA sequences, the pre-life Earth was capable of accurate complementary RNA replication. Very clearly, the first type II and type I tRNAs were generated by highly ordered, non-random processes on pre-life Earth.

Phylogenomics and bioinformatics methods provide insights into the first proteins and cofactors that coevolved with the genetic code [6,7,8,9]. Integrating these data with tRNA and genetic code evolution provides unprecedented insight into the pre-life to life transition on planet Earth.

A recent review describes a bricolage theory (multiple diverse functions coalescing into new functions) for the evolution of translation systems [10]. Our view would include a radially outward evolution component centered first on a primitive adapter molecule (ACCA-Gly) and then on RNAs of increasing complexity attaching 3′-ACCA-Gly, evolving by a recognized pathway to Gly-tRNA. We posit that dirty, pre-life polyglycine formed the core aggregator for the evolution of the earliest translation systems, protocells, and, after enrichment of the translation system, the first true cells. Our view posits a powerful chemical selection for the pre-life-to-life transition on Earth. The history that we relate is centered on tRNA and is based on the molecular history of tRNA, as inscribed in tRNA sequences. The mechanism for the evolution of tRNA demands that RNAs be ligated for complementary RNA replication. Thus, complex RNAs were generated during pre-life to supply many tasks of complex ribozymes and to generate the first rRNA-like molecules. So, an RNA World and a complex Peptide World radiated from tRNA. There does not appear to be a “chicken and egg” problem in the evolution of living cells on Earth. Our radially outward evolution model centered on tRNA makes rich predictions for many experiments.

We do not support the Gamow stereochemical model for the evolution of the genetic code, although an interesting version was recently published [11]. We consider any direct binding relationship between DNA codons and cognate amino acids to be a fanciful idea or else a losing strategy. For ideas about genetic code evolution other than ours, see references [12,13]. We consider a model in which a primitive adapter (ACCA-Gly) attaches to various RNAs of increasing complexity until attachment to tRNA, leading to the coevolution of tRNAomes, tRNA modifications, AARSomes, and the genetic code. We consider coding to have evolved first in tRNA. Through coevolution, the code in tRNA was complemented in mRNA. The code was later preserved in DNA.

## 2. Materials and Methods

Sequence logos are a visual way to relate multiple sequence data (https://weblogo.berkeley.edu/logo.cgi accessed on 19 January 2025) [14,15]. tRNA sequences were obtained from the tRNAdb [16] and gtRNAdb [17,18]. Unfortunately, the tRNAdb is currently offline, so some of its features used here may not be as easily reproducible as they otherwise would be. For instance, tRNAdb produced typical tRNA diagrams for an organism or domain with a few clicks of a mouse. Typical tRNA diagrams can be used to establish the three 31 nt minihelix tRNA evolution theorem using little effort [3,4].

Molecular structures were analyzed using ChimeraX (UCSF ChimeraX version 1.6.1) [19,20,21]. PDB 1EZH is tRNA^Phe^ (GAA) from *Saccharomyces cerevisiae* [22]. Although this is an eukaryotic tRNA, it has not changed much from an archaeal tRNA^Phe^. This is probably the best (i.e., highest resolution and best conformation) type I tRNA structure in the protein data bank. PDB 1WZ2 is from a tRNA^Leu^ (CAA)-LeuRS-IA co-crystal from *Pyrococcus horikoshii* (an ancient Archaeon) [23]. Because of the co-crystal structure, the anticodon loop of tRNA^Leu^ is somewhat unwound.

## 3. Evolution of Type II and Type I tRNAs

**Figure 1:**
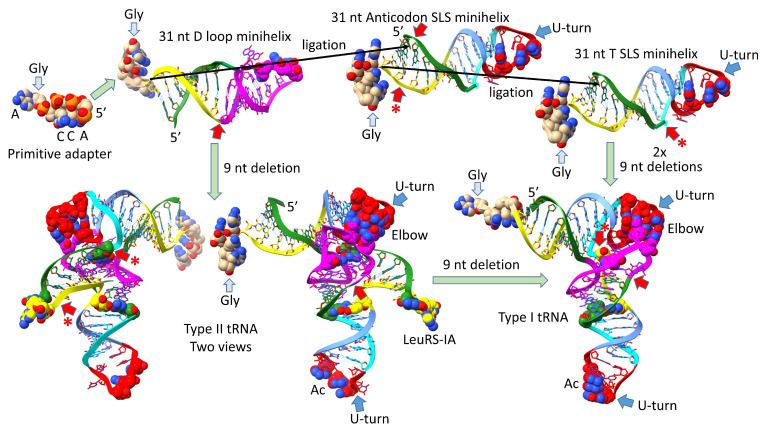
Evolution of type II and type I tRNAs from ligation of three 31 nt minihelices and internal RNA processing. ACCA-Gly was a pre-life adapter molecule that could function alone or ligated to various RNAs, including 31 nt minihelices and tRNAs. Black arrows indicate how ligations of three 31 nt minihelices generated the 93 nt tRNA precursor molecule that was processed into type II and type I tRNAs. In the 93 nt tRNA precursor, type II tRNAs were formed by a single internal 9 nt deletion within ligated 3′ and 5′ acceptor stems. Type I tRNAs were formed by an additional 9 nt deletion in the V arm region. The purpose of the initial molecules was to synthesize polyglycine. Colors: (green) 7 nt 5′-acceptor stems and 5 nt 5′-acceptor stem remnants; (magenta) 17 nt D loop core; (cyan-red-cornflower blue) 17 nt anticodon stem-loop-stem and homologous T stem-loop-stem; (yellow) 7 nt 3′-acceptor stem and 5 nt 3′-acceptor stem remnant (type I V loop). Red arrows indicate the sites of the 9 nt internal deletions. Red arrows with asterisks represent the more 3′-9 nt internal deletion unique to type I tRNA. Blue arrows indicate U-turns. Some bases are emphasized using space-filling representation.

**Figure 2:**
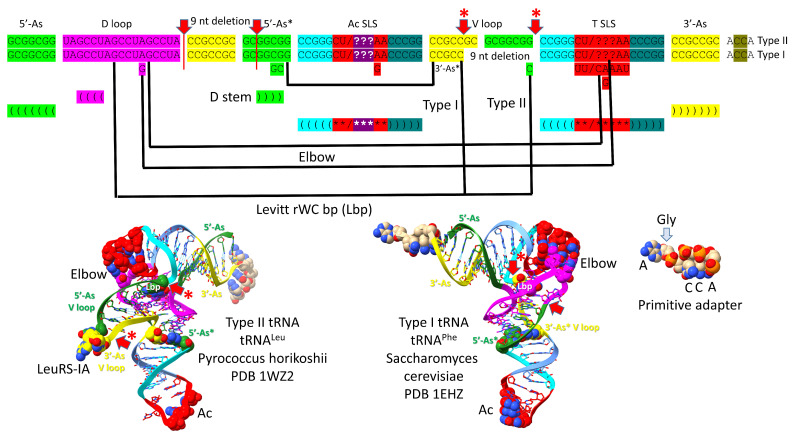
The pre-life 93 nt tRNA precursor was formed from ligation of three 31 nt minihelices (top sequence). Type II tRNA was formed by a single 9 nt internal deletion within ligated 3′- and 5′-acceptor stems. Type I tRNA was formed by an additional 9 nt internal deletion within ligated 3′- and 5′-acceptor stems (second sequence line). Interactions and sequence changes to support the tRNA fold are indicated. Red arrows indicate internal 9 nt deletion sites and endpoints. The 5′-9 nt deletion is common to type II and type I tRNAs. The closely related 3′-9 nt deletion was only for type I tRNAs. Red arrows with asterisks indicate processing of an early type II tRNA to a type I tRNA. Colors are consistent between figures. (As) indicates acceptor stem; (Ac) indicates anticodon; (Lbp) indicates the Levitt reverse Watson–Crick base pair. See the text for details.

The evolution of type II and type I tRNAs is summarized in Figure 1. The molecules are colored according to the three 31 nt minihelix tRNA evolution theorem, as published elsewhere [3,4] (see also Figure 2). A 31 nt D loop minihelix lacking the 3′-ACCA-Gly adapter was ligated to a 31 nt anticodon stem-loop-stem minihelix lacking the 3′-ACCA-Gly adapter, which was ligated to a second anticodon stem-loop-stem minihelix lacking the 3′-ACCA-Gly adapter or possibly to its very similar complement (to form the T stem-loop-stem) (Figure 1). The 93 nt tRNA precursor was then processed by two closely related internal 9 nt deletions within ligated 3′- and 5′-acceptor stems. An early version of the type II tRNA could have been processed by a single 9 nt internal deletion to a type I tRNA, so type II tRNA could have been a processing intermediate to type I tRNA, depending on the order of deletions to form type I tRNA [5]. The more 5′ internal 9 nt internal deletion was identical for both type II and type I tRNAs (red arrows). The two internal 9 nt deletions in type I tRNA were identical on complementary RNA strands. On pre-life Earth, the purpose of 31 nt minihelices and the first tRNAs was to synthesize polyglycine, posited to have been a main aggregator of pre-life intermediates and protocells [24,25]. The process for tRNA generation shows that the pre-life Earth was capable of the following: (1) RNA ligation (i.e., by a ribozyme RNA ligase); (2) complementary replication; (3) chiral sorting of precursors; (4) selection of 7 nt U-turn loops; (5) measuring stems and loops; (6) aminoacylation of ribose rings; (7) polypeptide synthesis; (8) internal processing of RNAs; and (9) sorting of U, A, G and C nucleotides and exclusion of other bases.

To correlate the tRNA structures to their root sequences, Figure 2 is shown. The entire tRNA sequence was formed from RNA repeats and inverted repeats (stem-loop-stems). Initially, the orderly process was a surprise because tRNA evolution had been proposed to have been chaotic [26,27,28,29,30,31]. The 7 nt 5′-acceptor stems and their 5 nt 5′-acceptor stem remnants were formed from 7 nt GCG repeats (GCGGCGG and after deletion GGCGG). The 3′-acceptor stems were formed from complementary 7 nt CGC repeats (CCGCCGC and, after deletion, CCGCC (the type I tRNA V loop)). The D loop was formed from a 17 nt UAGCC repeat. The anticodon and T stem-loop-stems had the initial 17 nt sequence ~CCGGG_CU/???AA_CCCGG. It is possible that the T stem-loop-stem initially formed from the complement of the anticodon stem-loop-stem (~CCGGG_UU/???AG_CCCGG). Ambiguity in the pre-life sequences arises because some of the nucleotides form the anticodon in tRNA, and the anticodon bases were scrambled in evolution to support coding. After LUCA (the last universal common (cellular) ancestor), the T loop sequence is known with confidence, but the T loop was strongly selected to form the tRNA “elbow”, where the D loop binds the T loop, so uncertainty about the pre-life 7 nt U-turn loop sequence (i.e., in 31 nt minihelices) remains at the anticodon base positions.

The major difference between type II and type I tRNAs is the variable loop (V loop). For type II tRNA, the sequence was initially a 3′-acceptor stem ligated to a 5′-acceptor stem (CCGCCGC_GCGGCGG) [5,32]. For type I tRNA, the V loop was initially CCGCC, processed from CCGCCGC_GCGGCGG by an internal 9 nt deletion removing GC_GCGGCGG. The type II tRNA V arm evolved to form a stem-loop-stem with a particular trajectory of the V arm from the tRNA body that is consistent within a synonymous tRNA set (i.e., in an Archaeon, there are 5 tRNA^Leu^ V arms with a common trajectory and similar sequence unique to tRNA^Leu^ and 4 tRNA^Ser^ V arms with a common trajectory and similar sequence unique to tRNA^Ser^). Type I V loops contact residues in the D stem, explaining differences in type I V loops from the original CCGCC sequence. The 5′-acceptor stem remnant (5′-As*) common to both type II and type I tRNAs was formed by an identical internal 9 nt deletion on the complementary RNA strand (CCGCCGC_GCGGCGG→GGCGG by deletion of CCGCCGC_GC). So, a single internal 9 nt deletion process can account for both the more 5′- and 3′-9 nt internal deletions in type I tRNA. Because of complementary replication in pre-life, the strands on which 9 nt deletions occurred cannot now be known.

To support the tRNA fold, some deviations occurred from the original tRNA sequences and are indicated in Figure 2. The Levitt reverse Watson–Crick base pair is indicated by tRNA-15G (or GD_8_) and the last base of the V loop (CV_5_ for type I tRNAs and CV_n_ for type II tRNAs). For type II tRNA V arms, the original sequence was GV_n_, but it was changed to CV_n_ to support the Levitt G=C base pair. A reverse Watson–Crick base pair is a standard Watson–Crick pair (i.e., in DNA) with one of the bases flipped over and shifted slightly. A G=C reverse Watson–Crick base pair forms two hydrogen bonds instead of three, as in DNA. tRNA-18G (GD_12_) replaced an A in the original UAGCC repeat sequence. GD_12_ intercalates between tRNA-57A or tRNA-57G and tRNA-58A and forms a hydrogen bond to tRNA-55U right before the T-loop U-turn. The A→GD_12_ sequence change supported the tRNA fold by stabilizing the tRNA elbow where the D loop and T loop interact. Another elbow contact is tRNA-19G (GD_13_), forming a Watson–Crick interaction with tRNA-56C. The 5′-As* sequence was slightly rearranged to support the stability of the D stem (typically 22-GGCGG-26→GGGCG to pair with D_3_-GCCU-D_6_). tRNA-G26 (or a substituted base) interacts with the V_1_ base. The anticodon loop was selected to support coding with various anticodon sequences (typically CU/BNNAA) (B is U, C or G and not A; N is A, G, C or U; / indicates the U-turn). The T loop (typically UU/CAAAU) was selected to support D loop contacts at the elbow.

## 4. The tRNA D Loop Was Based on a UAGCC Repeat

**Figure 3:**
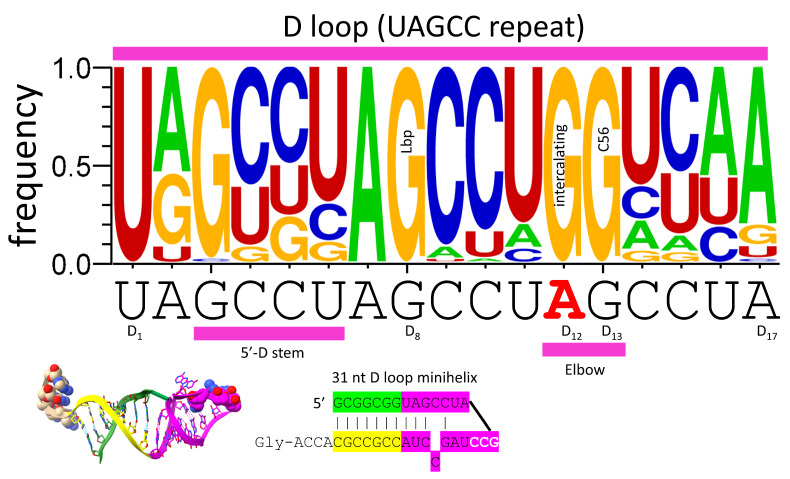
The 17 nt D loop core was based on a UAGCC repeat (UAGCCUAGCCUAGCCUA). The sequence and an approximate structure of the D loop minihelix are shown. Colors are meant to be consistent between figures. Magenta bars match segments in the structure and sequence below. GD_8_ forms the Levitt reverse Watson–Crick base pair to type I CV_5_ or type II CV_n_. Red AD_12_ was substituted by GD_12_ to form elbow contacts to the T loop. GD_13_ forms a Watson–Crick pair with tRNA-56C.

Figure 3 shows a sequence logo that demonstrates that the D loop of tRNA was formed from a 17 nt UAGCC repeat. GD_8_ interacts with CV_5_ in type I tRNA and CV_n_ in type II tRNA (In *Pyrococcus furiosus*, CV_14_ in tRNA^Leu^, and CV_15_ in tRNA^Ser^) to form the Levitt reverse Watson–Crick base pair. AD_12_ was substituted by GD_12_ to intercalate between tRNA-57A or -57G and tRNA-58A and hydrogen bond to tRNA-55U right before the T loop U-turn at the elbow. GD_13_ forms a Watson–Crick pair with tRNA-56C. The logos in this work were prepared from 46 *Pyrococcus furiosus* tRNAs, which represent a complete tRNAome set for an ancient organism. If the logos had been prepared from all Archaea, the results would not have been substantially different. We conclude that the D loop was derived from a UAGCC repeat.

## 5. The Anticodon and T Stem-Loop Stems

**Figure 4:**
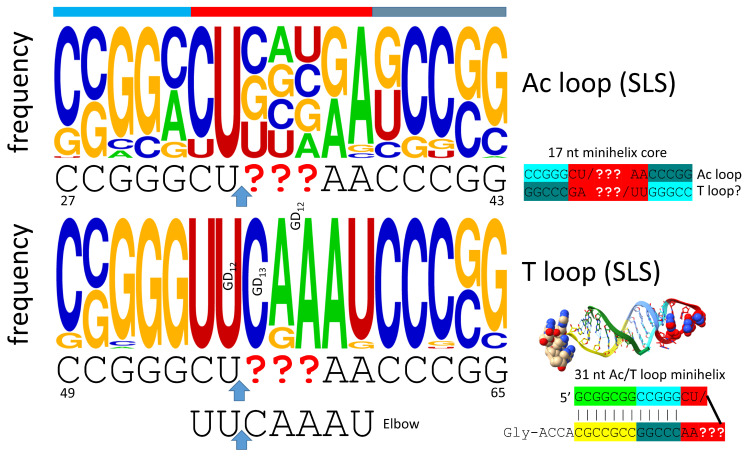
The anticodon (Ac) stem-loop-stem (SLS) and the T stem-loop-stem are homologs. The blue arrows indicate the position of the loop U-turn. In tRNA, the T loop (UU/CAAAU) evolved to interact with the D loop at the elbow. The T stem-loop-stem sequence is also very similar to the anticodon stem-loop-stem complement. Colors are meant to be consistent between figures. At the elbow: U55 interacts with GD_12_; GD_13_ binds C56 (Watson–Crick pair); GD_12_ intercalates between A57 or G57 and A58. The bar above the figure indicates the stem-loop-stem structure (cyan–red–cornflower blue).

Remarkably, the anticodon and T stem-loop-stems, as with the D loop UAGCC repeat, are also 17 nt in length (Figure 4). We conclude that the D loop functioned similarly to the anticodon and T stem-loop-stems on pre-life Earth, with a different sequence but a common purpose, which was synthesis of polyglycine. The anticodon stem-loop-stem evolved to support coding. In tRNA, the T stem-loop-stem evolved to support elbow contacts to the D loop. The anticodon stem-loop-stem has the top logo sequence CCGGC_CU/BNNGA_GCCGG. The T stem-loop-stem has the top logo sequence CCGGG_UU/CAAAU_CCCGG. Clearly, these are homologous sequences, simply by inspection. Because these are stem-loop-stems, they read very similarly on complementary strands. The T loop, therefore, may be derived from the complement of the anticodon stem-loop-stem, as indicated in Figure 4. We do not know, at this time, how to resolve which strand may have produced the T stem-loop-stem. The ~CU/BNNAA U-turn loop is significant intellectual property for the evolution of life, as described below. The anticodon/T stem-loop-stem minihelix is shown in Figure 4. This is a more rigid stem-loop stem than the D loop minihelix (Figure 3). On pre-life Earth, there may have been a negative selection against the rigid 31 nt anticodon stem-loop-stem minihelix with 12 C=G pairs (Figure 4). Difficulty in melting and replicating this long stem may have been part of the driving force for the evolution of tRNA, in which C=G stems are shorter and more easily melted. It is probable that the flexibility of the D loop minihelix contributed to the initial formation of tRNAs (Figure 3). That is to indicate that tRNA, perhaps, could not have evolved from the ligation of three anticodon stem-loop-stem minihelices, which might have been expected to be, instead, processed to three 31 nt anticodon minihelices.

At the base of genetic code evolution, wobble tRNA-34A was not utilized. Wobble tRNA-34G interacts with wobble mRNA-3C (Watson–Crick pairing) or -3U (wobble pairing), so wobble tRNA-34A was not necessary. Without modification, wobble tRNA-34C pairs well with mRNA-3G but poorly with mRNA-3A. tRNA-34U, therefore, was necessary to evolve the code, but unmodified wobble tRNA-34U was not ultimately suitable because of “superwobbling” [33,34,35]. In mitochondria, to shrink the size of the mitochondrial genome and tRNAome, a single unmodified wobble tRNA-34U reads wobble mRNA-3A, -3G, -3C and -3U. Evolving a code including two codon sectors, therefore, requires modification of tRNA-34U to restrict its reading. The acetyltransferase Elp3, which is as ancient as the genetic code, begins the modification [36,37]. For instance, tRNA-34cnm^5^U (5-cyanomethyluridine) is an example of a tRNA-34U 5-carbon modification initiated by Elp3, which may be as ancient as the genetic code to suppress superwobbling. The tRNA-34 wobble position, therefore, has only purine–pyrimidine resolution because reading A and U in a wobble position was awkward.

Reading A and U was awkward at wobble tRNA-34. Reading A and U was also awkward at tRNA-36. Notably, at the base of the code, if tRNA-36A was present, the tRNA-37m^1^G modification (or a similar modification) was also present. If tRNA-36U was present, the tRNA-37t^6^A modification (or a similar modification) was also present [33]. We conclude that modification of tRNA-37 affects the reading of tRNA-36, particularly if tRNA-36A or tRNA-36U is present. We conclude that tRNA-36 was most likely a wobble position at which wobbling was suppressed during code evolution, in part, by modification of tRNA-37. Note that wobbling at tRNA-34 could not be suppressed in the same manner as tRNA-36 because tRNA-33 is on the opposite side of the anticodon loop U-turn. Modification of tRNA-33U would be unlikely to affect the reading of tRNA-34 and might interfere with the U-turn conformation of the loop. Modification of tRNA-35 could not be performed to suppress tRNA-34 wobbling. As a Watson–Crick position for coding, there are four bases present at tRNA-35, and their modifications might disrupt coding.

## 6. The Anticodon Loop

**Figure 5:**
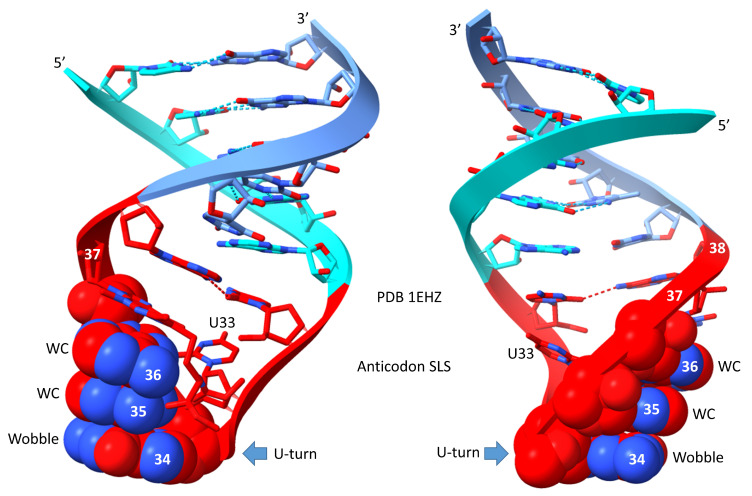
The anticodon stem-loop stem (two views). Colors and arrows are consistent between figures. WC for Watson–Crick.

The anticodon stem-loop-stem is shown in Figure 5. The anticodon loop (~CU/BNNAA) was indispensable for the evolution of tRNA and the genetic code. In the pre-life chemical evolution of life, the anticodon 7 nt loop with a U-turn between loop positions 2 and 3 was essential intellectual property. We posit that any attempt to replace this 7 nt U-turn loop with another RNA loop would have failed in evolution. An RNA loop of 3, 4, 5, 6, or 8 nt would certainly have failed. In the 7 nt U-turn loop, loop C1 interacts with loop A7 via a reverse Hoogsteen hydrogen bond. The interaction stacks the loop C1-A7 pair with the anticodon stem. Modifications to C1 (i.e., 2′-O-methyl-C; Cm) or A7 may affect this interaction [22]. The U-turn loop is a tight loop that would be expected to resist attacks by ribozyme nucleases in a pre-life world and, so, may have been strongly chemically selected. The C1-A7 pair and loop U2 set up the loop conformation to form the U-turn. The U-turn projects tRNA-34, tRNA-35, and tRNA-36 to form the 3 nt anticodon. Modifications of tRNA-37 contribute to the reading of tRNA-36. Apparently, wobbling at tRNA-36 was, in part, suppressed by modifications at tRNA-37. Wobbling at tRNA-34 cannot be suppressed in the same manner as tRNA-36, as described above.

## 7. Evolution of the Type II V Arm and Alignment to the Type I V Loop

**Figure 6:**
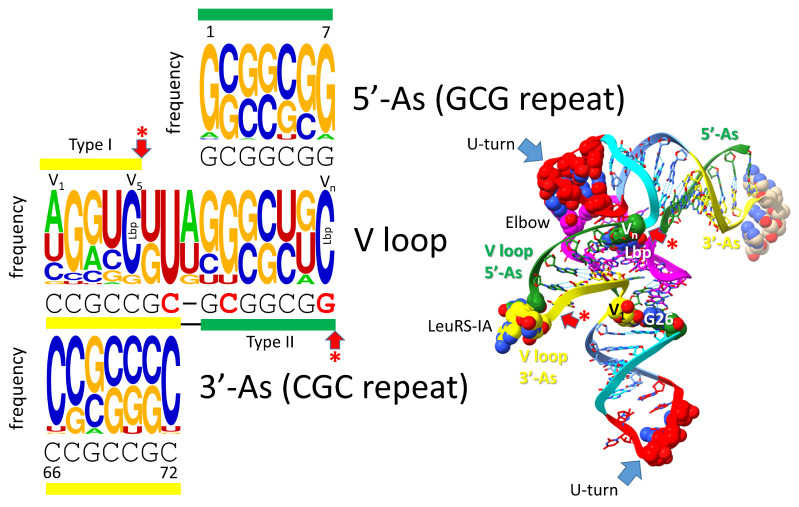
Evolution of the type II V arm and alignment to the type I V loop. The tRNA^Ser^ type II V arm single base insert may not be properly placed. Yellow (3′-acceptor stem) and green (5′-acceptor stem) bars indicate locations of sequences in the structure. tRNA^Leu^ and tRNA^Ser^ type II V arms evolved to form stem-loop-stems with different cognate sequences and trajectories that could be accurately discriminated by LeuRS-IA and SerRS-IIA. Red arrows with asterisks are the same as in other figures and, also, in the structure.

Figure 6 shows the mechanism of evolution of the tRNA type II V arm and how it aligns with the type I V loop [5,32]. The type II V arm was initially a 3′-acceptor stem (CCGCCGC) ligated to a 5′-acceptor stem (GCGGCGG) (see Figure 2). In the type II V arms, in an ancient Archaeon, UV_1_ is always present to interact with tRNA-G26. In an ancient Archaeon, CV_n_ is always present to form the Levitt reverse Watson–Crick base pair to tRNA-G15 (GD_8_). Because the original sequence (CCGCCGC_GCGGCGG) pairs along its entire length and can form tangled mispairs, the type II V arm evolved to form a stem-loop-stem. In Archaea, tRNA^Leu^ (5 synonymous tRNAs) and tRNA^Ser^ (4 synonymous tRNAs) have type II V arms. The number of synonymous type II tRNA sets in an organism was limited by the allowed trajectory set points of the type II V arms, described below [5]. Because LeuRS-IA and SerRS-IIA would have to read so many different cognate tRNAs with different anticodons, LeuRS-IA and SerRS-IIA utilized the projecting type II V arm as a determinant for accurate aminoacylation instead of the more variable anticodon loops. LeuRS-IA interacts with the tRNA^Leu^ type II V arm end loop (typically, V_6_-UAG-V_8_) [5,23,38]. SerRS-IIA binds the tRNA^Ser^ V arm stems and the elbow and recognizes the different trajectory of the tRNA^Ser^ V arms, rejecting the tRNA^Leu^ V arms [5,39]. In *Pyrococcus furiosus*, the type I V loop aligns with the first 5 nt of the type II V arms. Currently, type II V arms and type I V loops are improperly aligned in tRNA databases, and these alignments must be adjusted.

## 8. Evolution of the 3′-D Stem and Type I V Loop

**Figure 7:**
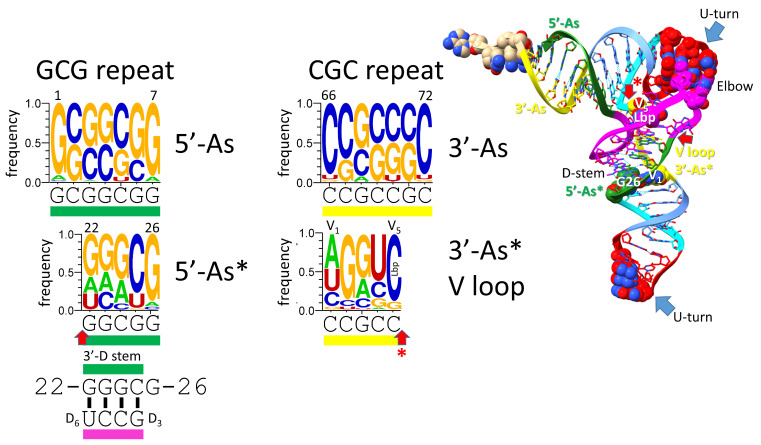
Evolution of the 3′-D stem (5′-As*) and the type I tRNA V loop (3′-As*). The color rectangles indicate the location of sequence within the structure. Arrows are consistent with other figures. Typical sequences of the D stem are indicated.

The 5′-As* green segment and the 3′-As* (type I V loop) yellow segment were formed by processing of ligated 3′- and 5′-acceptor stems (CCGCCGC_GCGGCGG) (Figure 7) (see also Figure 2). The 3′-D stem sequence was initially GGCGG. This sequence evolved typically to 22-GGGCG-26 in order to pair with D_3_-GCCU-D_6_ to form the 3′-D stem (see also Figure 2). tRNA-G26 (or a substitution) typically binds the V_1_ base. Statistical tests strongly support the homology of the 5′-acceptor stem and typical 22-GGGCG-26 5′-As* sequence, aligned as shown [3,32].

The type I V loop (3′-As*) initially had the sequence CCGCC but evolved typically to V_1_-AGGUC-V_5_ (Figure 7). V_1_ typically interacts with G26 or a substituted base. The type I V loop makes many interactions. GV_2_ can interact with GD_3_. GV_3_ forms a triplex base interaction with 22G that pairs with UD_6_ as part of the D stem. UV_4_ can flip away and not form interactions with other tRNA bases. These interactions are affected by tRNA modifications and sequences and may not be the same for every tRNA. Here, the PDB 1EHZ structure was considered [22]. CV_5_ forms the Levitt reverse Watson–Crick base pair to tRNA-G15 (GD_8_). Statistical tests strongly support the homology of the 3′-acceptor stem and 3′-As* (type I V loop), aligned as shown [3]. From the logos, the 5′-acceptor stem was clearly derived from a GCG repeat (typically GCGGCGG). The 3′-acceptor stem was clearly derived from its CGC repeat complement. It should be stressed that these clear sequence patterns and similarities have been preserved for ~4.2 billion years in tRNAs.

## 9. Evolution of the Type II V Arms and V Arm Trajectories

**Figure 8:**
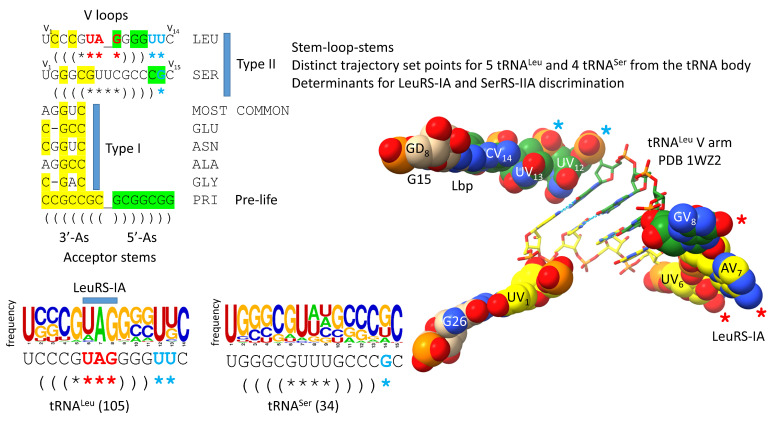
Alignment of type II V arms to type I V loops. The sequence alignment shows how type II V arms and type I V loops align with one another and their derivation from ligation of 3′- and 5′-acceptor stems. The tRNA^Leu^ (CAA) V arm of *Pyrococcus horikoshii* is shown [23,38]. Sequence logos of 105 14 nt archaeal tRNA^Leu^ V arms and 34 15 nt archaeal tRNA^Ser^ V arms are shown. In Archaea, tRNA^Leu^ and tRNA^Ser^ are discriminated by the following factors: (1) the distinct trajectory set point scores of the type II V arms (cyan asterisks); (2) the tRNA^Leu^ V_6_-UAG-V_8_ V arm-end loop sequence determinant for LeuRS-IA (red asterisks); and (3) SerRS-IIA binding the type II V arm stems of tRNA^Ser^ with a trajectory set point score of one unpaired base (cyan asterisk).

The number of synonymous tRNA sets in an organism that can utilize type II V arms is limited by the number of permitted V arm trajectories (Figure 8) [5]. In Archaea, only the synonymous sets of 5 tRNA^Leu^ and 4 tRNA^Ser^ utilize type II V arms. Each synonymous set of tRNA^Leu^ or tRNA^Ser^ has a common V arm trajectory, secondary structure and a similar sequence within the set. The V arm trajectory is given by the number of unpaired bases between the 3′-V arm stem and the Levitt base CV_n_. For tRNA^Leu^, the V arm has a trajectory set point score of two unpaired bases. For the tRNA^Ser^, the V arm has a trajectory set point score of one unpaired base. In Archaea, LeuRS-IA binds the tRNA^Leu^ V arm end loop V_6_-UAG-V_8_, which is highly conserved in Archaea (see the sequence logo). SerRS-IIA binds to the V arm stems and the elbow of the tRNA^Ser^. SerRS-IIA mostly recognizes the distinct trajectory of tRNA^Ser^ V arms and rejects tRNA^Leu^ V arms that have a cramped trajectory for SerRS-IIA binding contacts. In Archaea, only two type II V arm trajectories are observed. In Bacteria, by contrast, three type II V arm trajectories evolved, and bacterial tRNA^Tyr^ is also a type II tRNA. In Bacteria, for tRNA^Tyr^, the type II V arm has a trajectory set point score of two unpaired bases; tRNA^Leu^ has a trajectory set point score of one unpaired base; tRNA^Ser^ has a trajectory set point score of zero unpaired bases [5]. tRNA^Ser^ V arms in Bacteria are longer than in Archaea, which may explain, in part, the evolution of the third trajectory set point score of zero unpaired bases for bacterial tRNA^Ser^. The trajectory set point of 0 is not utilized in Archaea. In Bacteria, the longer tRNA^Ser^ V arm stems may stabilize the tighter connection at the base of the V arm stem (V_2_-V_(n-1)_).

## 10. ACCA-Gly Was the Pre-Life Adapter

**Figure 9:**
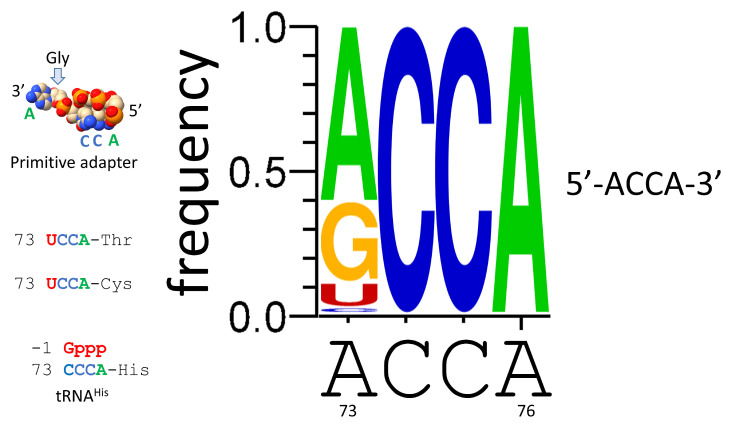
ACCA-Gly was the primordial adapter molecule. See the text for details.

We posit that ACCA-Gly was the primordial adapter molecule. After functioning alone, ACCA-Gly was ligated to other RNAs, including 31 nt minihelices and eventually tRNAs. RNAs in the pre-life world may have been modified at the 2′-O (i.e., 2′-O-methyl) to stabilize RNA against hydrolysis in a pre-life environment [40]. Figure 9 shows the last 4 nts of tRNA in *Pyrococcus furiosus* as a logo. tRNA-73 is considered the discriminator base because of its role in attaching the cognate amino acid by the cognate AARS enzyme. 74-CC-75 restrains the tRNA in the peptidyl and aminoacyl sites of the peptidyl transferase center of the ribosome. The ribose ring of tRNA-76A is the site of amino acid attachment. In an ancient Archaeon, tRNA-73A is the most common discriminator base. 73G is next, followed by 73U and 73C. In an ancient Archaeon, only tRNA^His^ utilizes discriminator tRNA-73C [16]. Because tRNA^His^ includes the sequence 73-CCCA, this may cause confusion in binding in the peptidyl site of the peptidyl transferase center of the ribosome. Confusion was relieved by modification of tRNA^His^ by posttranscriptional addition of GTP at the -1 position to pair with tRNA-73C. The addition of -1 GTP, which is specific for tRNA^His^, also provides a unique discriminator 73C=G(-1) pair to direct accurate and selective tRNA^His^ charging [41].

## 11. Polyglycine in Pre-Life

**Figure 10:**
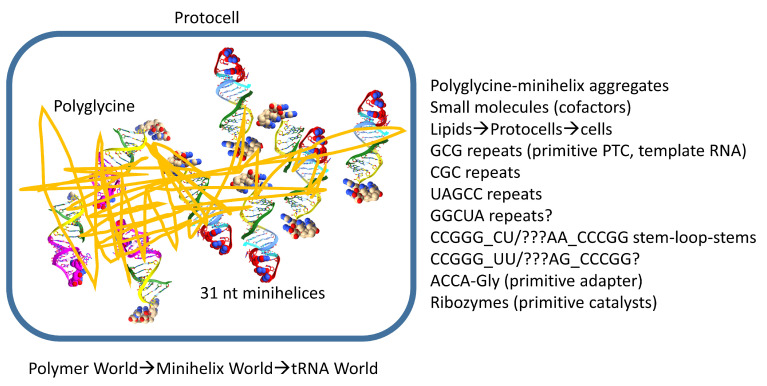
A proposed role for polyglycine in the pre-life world. Polyglycine is posited to have been the main aggregator of macromolecules that led to chemical selection, protocell enhancements, and formation of the first true cells. A list of some of the components aggregated by polyglycine that contributed to assembly of the first cells is shown. PTC for peptidyl–transferase center.

We posit that, in pre-life, polyglycine was initially the main aggregator of macromolecules, lipids, and cofactors that would eventually form the first cells (Figure 10). Thus, polyglycine was the major driving force for chemical selection and coevolution of tRNAomes and translation systems. Sequence analysis of tRNA evolution shows that Polymer World progressed to Minihelix World, which progressed to tRNA World [3,42]. Until Darwinian selection was imposed at about the time of LUCA, however, Polymer World, Minihelix World, and tRNA World coexisted because there was no straightforward mechanism for the destruction of competitors. At LUCA, the first cells could largely consume and destroy Polymer World, Minihelix World, and many other outmoded pre-life chemistries. Evidence for Polymer World and Minihelix World, however, endures and is embedded in the tRNA sequence. Sequences of tRNAs include GCG, CGC, and UAGCC repeats (Figure 2). Clearly, pre-life Earth produced RNA repeats. GCG and CGC repeats are complementary to one another, indicating accurate complementary replication of RNA on a pre-life Earth. Formation of stem-loop stems (i.e., ~CCGGG_CU/???AA_CCCGG) also indicates complementary replication on pre-life Earth because a stem-loop-stem can act as a snap-back primer for complementary replication. ACCA-Gly is posited to be the primitive adapter molecule attached to RNAs on pre-life Earth (Figure 9). Ribozymes supported necessary catalysis. Polyglycine could be enriched as a primitive aggregator through interactions with the pre-life environment (i.e., ultraviolet light, amino acid incorporation errors (innovations), and other chemical reactions) (Figure 10).

## 12. A Model for Evolution of the First Cells

**Figure 11:**
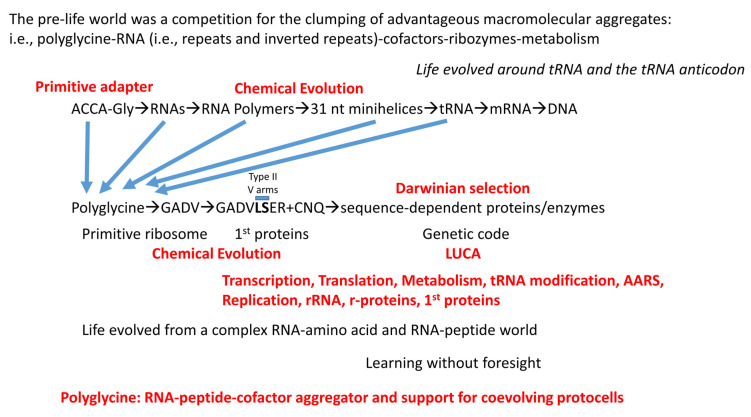
A working model for chemical evolution of the first cells. See the text for details.

**Figure 12:**
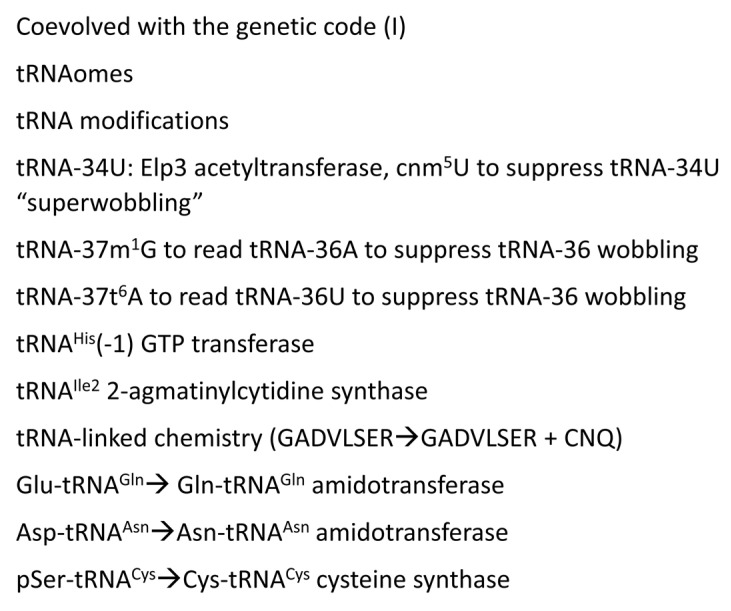
tRNAomes, tRNA modifications, and tRNA-linked reactions that coevolved with the genetic code.

**Figure 13:**
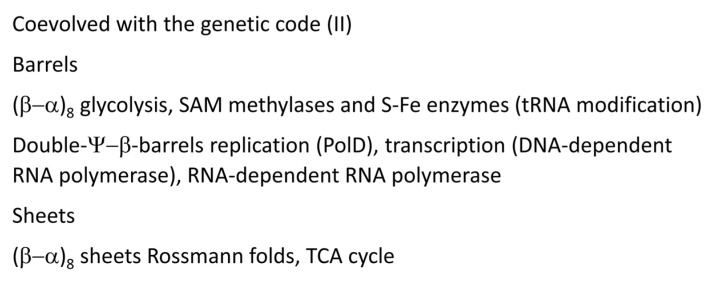
Protein barrels and sheets coevolved with the genetic code.

**Figure 14:**
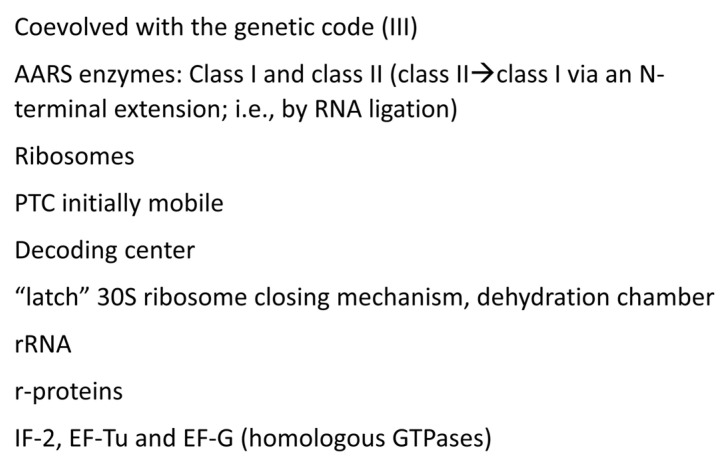
AARS, ribosomes, and translation factors coevolved with the genetic code.

A model to describe the chemical evolution of the first cells is shown in Figure 11. There is no “chicken and egg” problem in the pre-life-to-life transition, and no supreme deity appears to be required. Phylogenomics and bioinformatics provide insight into the enzymes and cofactors that were likely present at LUCA [6,7,8,9,43,44,45]. Here, we attempt to correlate parts of that emerging analysis with inferences that are based on tRNA evolution, as recorded in conserved tRNA sequence. Figure 12, Figure 13 and Figure 14 provide lists of some of the tRNA modifications and protein enzymes identified for LUCA that we infer must have coevolved with the genetic code.

Life on Earth evolved around tRNA and the tRNA anticodon (Figure 11). So far as we can see, this conclusion cannot now rationally be questioned. Translation systems and the genetic code coevolved with tRNAomes. Without a genetic adapter as good or better than tRNA, it is difficult to consider how else complex encoded enzymes and proteins could have evolved. Considering structure, function, and evolution, it is difficult to re-design tRNA to evolve a more advantageous genetic adapter. Replacing tRNA with a genetic adapter that is not comprised of RNA and that has not evolved within an aqueous environment is a daunting problem. Altering the tRNA anticodon loop to another loop structure (i.e., 3, 4, 5, 6, or 8 nt) is also a daunting problem to which there may not be a reasonable solution. The 7 nt U-turn tRNA anticodon loop, which was powerfully selected in pre-life, must evolve to a 3 nt genetic code with a single tRNA-34 wobble position (Figure 5). The chances of evolving to a 4 nt genetic code are vanishingly slim. Ribosomes evolved around tRNA. For instance, the large ribosomal subunit includes a tRNA-shaped channel through which the tRNAs advance [46]. We conclude that the genetic code and sequence-dependent proteins evolved around tRNA and the tRNA anticodon (Figure 11). Also, mRNA evolved secondarily to tRNA, so the evolution of the tRNA anticodon directed mRNA codon evolution. Therefore, the evolution of tRNA is the central story in the evolution of life on Earth. Fortunately, the history of tRNA evolution was recorded and conserved for ~4.2 billion years in tRNA sequence.

We posit two coupled mechanisms for aggregation of pre-life macromolecules leading to life: (1) aggregation of macromolecules interacting with (dirty) polyglycine and (2) progression of lipids→protocells→cells probably emulsified by polyglycine. When we refer to polyglycine, we consider dirty polyglycine with other induced chemistries (i.e., reaction products from ultraviolet light exposure) and associations (i.e., binding by other amino acids and chemicals). Glycine was probably the first encoded amino acid. Glycine is the simplest amino acid. Glycine occupies the most favored sector in the genetic code (tRNA^Gly^ (BCC)), indicating that glycine might be the first encoded amino acid [24,25,42,47,48]. Glycine was present on pre-life Earth.

We posit ACCA-Gly as a primitive adapter molecule (Figure 9). We posit that ACCA-Gly was ligated (i.e., by a ribozyme ligase) to many RNAs on pre-life Earth (Figure 11). Analysis of the tRNA sequence reveals that GCG, CGC, and UAGCC repeats were present in pre-life Earth. Also, ~CCGGG_CU/???AA_CCCGG stem-loop-stems were present. Multiple ACCA-Gly could assemble on GCGGCGGCG repeats (as in 5′-acceptor stems). The binding of multiple ACCA-Gly in proximity should be sufficient to support polyglycine synthesis (i.e., with wet–dry cycles) [40].

In the progression of RNAs of increasing complexity, GCG repeats, CGC repeats, UAGCC repeats, and stem-loop-stems were recombined into 31 nt minihelices (Figure 11). With ACCA-Gly attached, these 35 nt minihelices could have been used to synthesize polyglycine using a coevolved and mobile peptidyl transferase center that may first have arisen from GCG repeats. At some point, a primitive decoding center and the first mRNA-like molecules coevolved. We posit that polyglycine synthesis was the primary selective chemical driving force. As a molecular aggregator, polyglycine provided the major selection for pre-life chemistries leading to the first cells. As noted above, 31 nt minihelices may have been partly negatively selected against because of their long and very stable stems. This negative selection may have provided some of the impetus to evolve the first tRNAs, which did not include the longer stems that may have rendered minihelices difficult to unwind and replicate. Also, tRNA was positively selected because it is a molecule that can “teach” itself to code by duplication and repurposing in a pre-life environment.

We posit that the initial purpose of type I and type II tRNAs was to synthesize polyglycine on a primitive decoding center with a primitive mRNA, utilizing a mobile, primitive peptidyl transferase center perhaps derived initially from GCG repeats. As reported elsewhere, the genetic code appears to have evolved from encoding G→GADV→GADVLSER+CNQ→20 aas plus stops [3,5,42,47,48] (Figure 11). The coevolution of tRNAomes, AARS, ribosomes, mRNA, tRNA-linked chemistry, first proteins, and protocells drove the evolution of the first cells. We define the first proteins as those that coevolved with the genetic code. We consider proteins identified at LUCA by phylogenomics studies to be likely candidates for first proteins.

We posit that the tRNA-based genetic code initially evolved to synthesize polyglycine. GADV are the four simplest amino acids and are posited to be the first four encoded amino acids [49,50,51,52,53,54]. GADV occupies the most favored row in the evolution of the genetic code (tRNA-36C; anticodon BNC). The genetic code then appears to have progressed to an eight-amino-acid bottleneck (i.e., GADVLSER). The eight-amino-acid bottleneck arose because both tRNA-34 and tRNA-36 were wobble positions, and only a single wobble position could be read at a time [3,5,42,48]. At a wobble position, only pyrimidine–purine resolution could be achieved, so, at this stage, the maximum complexity of the code was 2 * 4 or 4 * 2, which equals 8. Leucine and serine evolved to occupy six-codon sectors in the final code, and tRNA^Leu^ and tRNA^Ser^ are type II tRNAs with longer V arms for cognate LeuRS-IA and SerRS-IIA recognition to avoid anticodon ambiguity in discrimination. Arginine also occupies a six-codon sector of the code, but tRNA^Arg^ is a type I tRNA. To circumvent coding ambiguity, ArgRS-IA unwinds the tRNA^Arg^ anticodon loop to expose additional bases for cognate tRNA^Arg^ recognition and charging [55]. It is likely that the strategy for cognate charging of tRNA^Arg^ (unwinding the anticodon loop) could not have supported three six-codon boxes for leucine, serine, and arginine. Preservation of type II V arms for tRNA^Leu^ and tRNA^Ser^, therefore, was likely necessary for the evolution of the code. Put simply, two strategies (type II V arms with distinct trajectory set points (tRNA^Leu^ and tRNA^Ser^) and unwinding of the anticodon loop (tRNA^Arg^)) to support six-codon boxes evolved because two different strategies were required. CNQ may have been added to the expanding code via tRNA-linked chemistry. Serine→cysteine [56,57], aspartic acid→asparagine, and glutamic acid→glutamine [58,59] are reactions identified in ancient organisms today. We posit that these reactions expanded the 8-amino-acid bottleneck to an 11-amino-acid code that could support the synthesis of the first proteins to coevolve with the code. Many proteins essential for life and conserved to the present day coevolved with the genetic code. Upon suppression of wobbling at tRNA-36, the genetic code expanded to 20 amino acids plus stops. Because wobbling at tRNA-34 limits coding to purine versus pyrimidine discrimination, the maximum complexity of the genetic code is 2 * 4 * 4 = 32 assignments. Wobbling at tRNA-34 could not be suppressed in evolution. The code froze at 20 amino acids plus stops because of fidelity mechanisms. In textbooks and school, the genetic code is described as having a complexity of 4 * 4 * 4 = 64 assignments. That is not correct because the purine-pyrimidine resolution of wobble tRNA-34 on the ribosome requires degeneracy.

Figure 12, Figure 13 and Figure 14 describe some of the enzymes and RNAs that coevolved with the genetic code to indicate how biological complexity arose [6,7,8,9,45]. Figure 12 concentrates on tRNAomes, tRNA modifications, and tRNA-linked chemistry supporting code evolution. We posit that LUCA had a fully established genetic code [6,7]. tRNAomes coevolved with mRNA, ribosomes, AARS enzymes, and other first proteins. tRNA modifications were necessary to evolve the code. Specifically, tRNA-34U 5-carbon modifications (i.e., tRNA-34cnm^5^U) were necessary to suppress superwobbling [33]. Such modifications begin with the Elp3 acetyltransferase. The five-carbon tRNA-34U is acetylated by Elp3, followed by additional modifications [36,37]. tRNA-34cnm^5^U appears to be one of the oldest such modifications. Without suppression of superwobbling, the genetic code would lack two-codon boxes. Reading tRNA-36A required a tRNA-37m^1^G modification or a variation. To read tRNA-36U required a tRNA-37t^6^A modification or a variation. We posit that tRNA-36 was initially a wobble position. Wobbling at tRNA-36 was partially suppressed by tRNA-37 modifications. tRNA^His^(-1) GTP transferase was necessary to properly position peptide-tRNA^His^ in the peptidyl site of the peptidyl transferase center of the ribosome during translation (Figure 9). tRNA^His^(-1) GTP transferase also confers a unique discriminator sequence for accurate histidine charging by HisRS-IIA [36,60]. For methionine to enter the genetic code, tRNA^Ile2^ 2-agmatinylcytidine synthase and loss of tRNA^Ile^ (UAU) were required (Figure 12). We posit that the evolution of the genetic code stalled at eight amino acids because of wobbling at tRNA-36. The eight-amino-acid bottleneck was partially relieved by (1) modifications at tRNA-37 [33]; (2) tRNA-linked chemistry to synthesize C, N, and Q from S, D, and E; and (3) evolution of the decoding center “latch” (see below). S→pSer→C reactions have been characterized [57]. D→N and E→Q amidotransferases have been identified [58,59,61].

Figure 13 indicates protein barrels and sheets that support metabolism, DNA replication, transcription, and essential tRNA modifications. We have proposed that (β–α)_8_ barrels (i.e., TIM barrels; TIM for triosephosphate isomerase) were formed by a similar mechanism to tRNA in which multiple similar or identical RNAs were ligated for replication (Figure 1) [3]. Ligation of multiple βαβα encoding units resulted in (β–α)_8_ barrels, which were also refolded into (β–α)_8_ sheets (losing β7 in refolding). TIM barrels and Rossmann folds describe much of core metabolism. Double-Ψ–β barrels were formed similarly to (β–α)_8_ barrels by RNA ligation, in this case, from two RNAs encoding ββαβ units followed by folding into the barrel ββαβββαβ. Two double-Ψ–β-barrel-type enzymes describe DNA polymerase PolD in Archaea, which may be the first replicative DNA-dependent DNA polymerase [43,62] and also DNA-dependent RNA polymerases in all organisms [2,43].

A summary of additional first protein translation functions is shown in Figure 14. Ribosomes are posited to have evolved from an independent decoding center and peptidyl transferase center. Translation initiates on the small ribosomal subunit, which includes the decoding center and most of the “latch”. The latch enforces Watson–Crick geometry at the two Watson–Crick positions (anticodon tRNA-35 and -36; mRNA codon 1 and 2) and regulates wobbling (anticodon wobble tRNA-34; mRNA wobble codon 3) [63,64,65,66,67]. The ribosomal large subunit that includes the peptidyl transferase center (aminoacyl site and peptidyl site) couples with the small subunit after initiation, and, in tandem, the small and large ribosomal subunits support accurate stepwise translocation. Initiation factor 2 (IF-2), EF-Tu, and EF-G are homologous GTPases that support initiation, aa-tRNA entry and translocation. EF-Tu and EF-G alternate binding to shared, overlapping sites mostly on the large ribosomal subunit during elongation. We consider the translation system to be relatively simple in concept but complex in its genetics and evolved structure. Basically, the ribosome appears to be a spare initial assembly with a complex genetic history and many add-ons to the original evolving functional core.

Recent work on ancient protein folds SH3 and OB that are common in ribosomal proteins and, also, cradle-loop barrels (i.e., double-Ψ–β-barrels; Figure 13) is very consistent with mechanisms we describe for the evolution of first proteins [68,69].

LUCA appears to have encoded a full set of AARS enzymes and, therefore, must have had an intact genetic code. Despite claims to the contrary [70,71,72,73], class II and class I AARS enzymes are simple, direct homologs [42,47,48,74]. Apparently, the first class I AARS (probably a primitive ValRS-IA) was formed by the addition of an N-terminal extension to a primitive GlyRS-IIA. From the tRNA evolution scheme, probably, the 5′-RNA N-terminal encoding extension was ligated to a GlyRS-IIA RNA for replication, much as described above for 31 nt minihelix replication and tRNA evolution (Figure 1). Because translation termination was imprecise, these RNAs need not have necessarily been initially ligated in phase.

## 13. Order of Addition of Amino Acids into the Genetic Code

The precise order of addition of amino acids into the genetic code is important but has been challenging to determine. Recently, phylogenomic studies have been used to attempt to make this determination [7]. In contrast, we have approximated an order of addition into the code from inference based on the highly structured code [5,42,47,48]. We find the phylogenomic arguments somewhat awkward in providing a clear answer to this fundamental question about the origin of life. Also, we experienced some difficulty in sorting different orders of amino acid addition that arise from different methods of ascertainment. What we conclude is that the best current understanding of this issue results if the order of amino acid additions is split into different columns of the genetic code, which causes different determinations to make better sense and to approach closer agreement.

**Figure 15:**
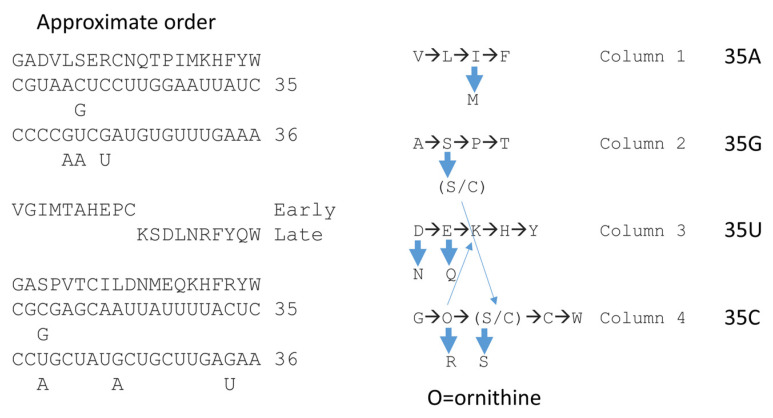
Splitting amino acids by genetic code columns helps to explain the order of addition of amino acids into the code. In the approximate order, we provide our determinations and two versions by another group [7]: (1) early and late additions and (2) another determination based on comparisons of amino acid usages at pre-LUCA and LUCA. tRNA-35 and tRNA-36 anticodon bases are indicated. Breaking the genetic code into code columns (tRNA-35) causes the information to make better sense. Blue arrows indicate derivations of one amino acid from another.

**Figure 16:**
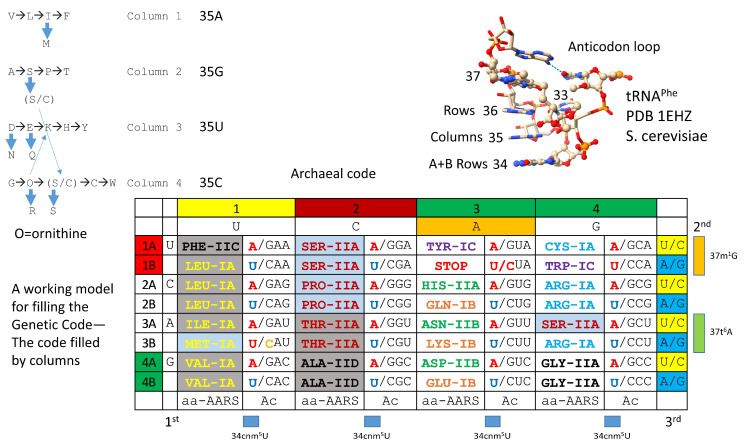
Correlation of amino acid additions with the archaeal genetic code. AARS enzymes are colored to emphasize patterns of evolution within code columns. Grey shading indicates an AARS editing active site that is separate from the aminoacylating active site. Blue shading indicates an editing reaction within the AARS aminoacylating active site. Wobble tRNA-34 bases shown in red are not utilized in Archaea. tRNA-34U in blue indicates that the Elp3 acetyltransferase-initiated modification (i.e., tRNA-34cnm^5^U) was necessary to suppress superwobbling. tRNA-37m^1^G was necessary to read tRNA-36A. tRNA-37t^6^A was necessary to read tRNA-36U. To encode isoleucine, CAU was used with C modified to agmatidine. To encode methionine, CAU was used with C unmodified (initiation) or C lightly modified (elongation; Cm). The structure of the code requires several first proteins coevolved with the code.

Figure 15 and Figure 16 describe our best current understanding of this issue presented as a working model for amino acid entry. Figure 15 shows how consideration of evolution within code columns can simplify the discussion. Very clearly, the genetic code evolved to a large extent within code columns around the tRNA-35 position. Glycine is the simplest amino acid and occupies the most favorable anticodon (tRNA-35C, tRNA-36C) [3]. The simplest amino acids, GADV, are found on the fourth row of the code (tRNA-36C), indicating that these were the first amino acids to be encoded [49,50,51,52,53,54]. It appears that amino acids entered the code by filling larger sections, which were then reduced in size as other amino acids entered. The amino acids that entered first, ultimately, retained the most favored available anticodons, according to the rules tRNA-35 (C>G>U>A) and tRNA-36 (C>G>U>>A).

In Figure 16, the working model for amino acid additions is correlated with the structure of the archaeal genetic code, which, we posit, was the code at LUCA [5,42,47,48,74]. The bacterial and eukaryotic codes appear to be derived from the more primitive archaeal code at LUCA and via fusions at LECA (the last eukaryotic common ancestor). In genetic code column 1, VIML are similar hydrophobic amino acids, and ValRS-IA, IleRS-IA, MetRS-IA, and LeuRS-IA are closely related AARS enzymes. Methionine is posited to have invaded a four-codon isoleucine sector, leading to differential wobble C modifications (to encode isoleucine, C→agmatidine [75,76]; to encode methionine, C is lightly modified (elongation) or unmodified (initiation)). Also, anticodon UAU is not utilized at the base of the code because the use of UAU would cause ambiguity in coding for isoleucine and methionine. Leucine eventually occupies a six-codon sector. We posit that an enlarged leucine sector gave rise to invasion by isoleucine and then methionine.

In column 2, ATPS are neutral amino acids. T and S are chemically related. Serine eventually occupies a six-codon sector of the code that is split between column 2 and column 4. Serine is the only amino acid that splits between two code columns. ThrRS-IIA, ProRS-IIA, and SerRS-IIA are closely related AARS enzymes. We posit that a now-extinct AlaRS-IIA may have been replaced before LUCA with AlaRS-IID to suppress tRNA charging errors. We posit that an enlarged serine sector gave rise to threonine and proline sections and also may have allowed for early entry of cysteine into the code. Cysteine, for instance, was necessary for the early folding of AARS enzymes by binding Zn [74]. We posit that serine jumped from column 2 to column 4 of the genetic code, and this event may be associated with the early entry of cysteine into the code. Cysteine can be generated from serine by two mechanisms [56,57]. In a later event, cysteine ended up on column 4, row 1 of the code. Row 1 of the code was initially disfavored because tRNA-36A did not function well in a wobble position before the suppression of wobbling at tRNA-36.

We have previously suggested that D and E entered the code to form a striped pattern in column 3 that resolved to D, N, and H in rows 4A, 3A, and 2A and E, K, and Q in rows 4B, 3B, and 2B. In tRNA-linked reactions, D→N and E→Q reactions were supported by amidotransferase enzymes [58,59,61]. Note that, in Archaea, AspRS-IIB (row 4A), AsnRS-IIB (row 3A), and HisRS-IIA (row 2A) are closely related AARS enzymes. In Archaea, GluRS-IB (row 4B), LysRS-IB (row 3B), and GlnRS-IB (row 2B) are closely related AARS enzymes. Interestingly, GlnRS-IB was not utilized at the base of code evolution. GlnRS-IB was generated in eukaryotic systems and acquired in archaeal systems via horizontal gene transfers [33]. At the base of code evolution, a dual function GluRS-IB was coupled with the Glu-tRNA^Gln^ amidotransferase to generate Gln-tRNA^Gln^. In Bacteria, LysRS-IIB replaced LysRS-IB in Archaea. LysRS-IB in Archaea is posited to be the older LysRS.

Column 4 of the code was the most favored column (tRNA-35C), explaining why glycine, the first encoded amino acid, occupies column 4, row 4 (BCC anticodon; the most favored anticodon) (Figure 16). Arginine occupies a six-codon sector of the code that was invaded by serine. It may be that ornithine was the initial positively charged amino acid that entered the code [77]. Ornithine can be converted to arginine in two steps. Ornithine is flexible, similar to lysine. Arginine is more rigid and forms strong ion pairs to aspartic acid, which are formed and broken in allosteric switching for many enzymes and proteins. Lysine entry into column 3 may relate to ornithine having been present in column 4 (i.e., initially, only a single CCU→CUU anticodon base change may have been required for ornithine or lysine jumping from column 4 into column 3).

Aromatic amino acids, FYW, are posited to have added late, across disfavored row 1 of the genetic code, perhaps initially as a now-extinct PheRS-IC AARS [5,42,47]. We posit that PheRS-IC was replaced by PheRS-IIC before LUCA to discriminate phenylalanine and tyrosine that utilizes TyrRS-IC, which is closely related to TrpRS-IC.

When considered according to genetic code columns, our working model closely relates to the orders of addition proposed by others. Our model stresses the importance of coupling metabolism to tRNAome and genetic code evolution. For instance, multiple tRNA-linked metabolic reactions can be identified in code evolution. S→C, D→N, and E→Q could be attributed to tRNA-linked chemistry. O→R (O for ornithine), F→Y, and V→L may be other examples of tRNA-linked reactions in the evolution of the code. In pre-life, metabolism and genetic code evolution were tightly coupled. As soon as isoleucine was encoded, methionine could be incorporated into the code. We posit that arginine, which occupies a six-codon sector, entered earlier than others proposed. This discrepancy may relate to the posited replacement of ornithine by arginine and the enhanced roles of arginine in allosteric shifts in sequence-dependent proteins.

Methionine occupies a one-codon sector because methionine invaded a four-codon isoleucine box. Differential modifications of wobble tRNA-34C to agmatidine (isoleucine) or 2′-O-methyl-C (methionine; elongation) and elimination of the UAU anticodon tRNA describe these events. The isoleucine sector was reduced to a three-codon box. Tryptophan occupies a one-codon sector and shares a two-codon box with a stop codon. Stop codons do not utilize a tRNA and are recognized instead as stop codons bound by protein release factors that interact with mRNA on the ribosome to terminate the reading frame [78]. Splitting a two-codon sector into two different amino acids presents problems that have not been solved in evolution.

## 14. Pre-Life RNA Replication Mechanism Generated Complexity

At the time of pre-life tRNA evolution, RNA was replicated by the following: (1) RNA ligations [79,80]; (2) ligating snap-back primers (stem-loop stems); and (3) complementary replication (mechanism unknown) [3,81,82,83]. RNAs were processed from large, complex RNAs by ribozyme endonucleases [84]. Because of the replication and processing mechanisms, similar or identical RNAs (i.e., 31 nt minihelices) accumulated. Replication joined similar and identical RNAs, generating RNA repeats. tRNA was generated from the ligation of a D loop 31 nt minihelix to two anticodon 31 nt stem-loop-stem minihelices (Figure 1). We posit that complex RNAs, such as rRNAs, were initially generated by a similar mechanism. Translation of RNA repeats generated protein barrels and protein linear sheets refolded from barrels. Ligation of different RNAs generated complex RNA recombinants that could provide new protein folds and functions. SH3 and OB folds and cradle-loop barrels (i.e., double-Ψ–β barrels) (Figure 13) were likely generated in this manner [68,69]. ValRS-IA (a class I AARS) was initially generated by attaching a 5′-RNA (origin unknown) to a 3′-GlyRS-IIA (a class II AARS) RNA [3]. The replication mechanism of ligations and processing accelerated pre-life complexity, and many enduring and diverse RNA and protein functions were generated. Because complex molecules could be synthesized early in pre-life, it is generally unnecessary to insist that simple RNAs and simple proteins (i.e., “urzymes”) [70,71] had to precede more complex forms. Significant molecular complexity was generated very early on pre-life Earth.

## 15. Discussion

Sequence logos colorfully illuminate the three 31 nt minihelix tRNA evolution theorem. tRNA evolved by ligation of three 31 nt minihelices of mostly known sequence to form a 93 nt tRNA precursor (Figure 1 and Figure 2). RNA ligations were necessary to support the RNA replication mechanism on pre-life Earth, resulting in complex and diverse pre-biology. In order to generate tRNA, internal 9 nt deletions were required within the 93 nt tRNA precursor RNA where 3′- and 5′-acceptor stems were ligated. An early version of type II tRNA could have been processed to type I tRNA by a single internal 9 nt deletion within ligated acceptor stems (Figure 1 and Figure 2). The two 9 nt internal deletions to generate type I tRNAs were identical on complementary RNA strands. Also, RNA repeats and inverted repeats conserved in tRNA sequences were generated on pre-life Earth. The 7 nt U-turn anticodon loop was selected in pre-life, and competing loops were negatively selected because of sensitivity to ribozyme nucleases. The structure of the anticodon loop requires an evolution of a 3 nt genetic code with a single wobble position. tRNA evolution demonstrates how highly ordered chemical evolution processes generated some of the first complex macromolecules, leading to the chemical evolution of tRNAomes, AARS enzymes, first proteins, the genetic code, rRNA, ribosomes, DNA genomes, and complex cells.

## 16. Conclusions

Type I and type II tRNAs evolved chemically via highly ordered mechanisms during pre-life. Very likely, these steps could be reproduced in laboratories.

The mechanisms for chemical evolution of tRNAs can be extended to generate highly complex RNAs such as rRNAs.

Polyglycine is proposed to have been a primary aggregator of pre-life macromolecules and cofactors and also to have promoted the transition of lipids to protocells and protocells to cells. The utility of polyglycine in promoting these chemistries could be reproduced in laboratories.

With the coevolution of translation functions, mechanisms for the evolution of the first proteins that coevolved with the genetic code can be described. RNA ligations of similar or identical RNAs generated barrels. Refolding of barrels generated linear sheets (i.e., Rossmann folds). Class I AARS were initially generated by attachment of an N-terminal extension to a primitive GlyRS-IIA (i.e., by RNA ligation for replication).

A straightforward and rational working model for the evolution of the genetic code has been proposed based initially on the chemical evolution of tRNA and the tRNA anticodon (Figure 15 and Figure 16). The model is supported by the coevolution of tRNAomes and AARS enzymes. Strong predictions arise about the order of entry of amino acids into the code and also the positioning of amino acids in the code. tRNA-linked reactions were necessary for code evolution. Metabolism, tRNAome, and code evolution were tightly coupled. Evolution was primarily within code columns. There was very little chaos in the chemical evolution of the code.

By focusing on the prominence of tRNA in the evolution of translation systems, the genetic code, and life, we emphasize the central winning pathway. Our approach has been a top–down, sequence-based approach, so the evidence for our conclusions is largely embedded in tRNA sequences in living organisms. By contrast, a bottom–up approach would be to create life in a test tube from pre-life components. Much very reasonable pre-life chemistry performed in laboratories, however, may represent dead-end strategies. Pre-life chemistry must have been complicated and diverse, and many chemically evolved processes must have gone extinct with the first organisms around the time of LUCA (i.e., Polymer World and Minihelix World mostly went extinct). tRNA was a molecule evolved and chemically positively selected in pre-life that has survived, supporting life for ~4.2 billion years.

## Data Availability

No new data were created or analyzed in this study.
